# Association between the platelet/high-density lipoprotein cholesterol ratio and nonalcoholic fatty liver disease: results from NHANES 2017–2020

**DOI:** 10.1186/s12944-023-01861-9

**Published:** 2023-08-11

**Authors:** Chun-feng Lu, Xiao-min Cang, Wang-shu Liu, Li-hua Wang, Hai-yan Huang, Sheng-mei Sang, Xue-qin Wang, Xing-xing Fang, Feng Xu

**Affiliations:** 1 Department of Endocrinology, Affiliated Hospital 2 of Nantong University and First People’s Hospital of Nantong City, No. 6 North Hai-Er-Xiang Road, Nantong, 226001 China; 2 Department of Nursing, Affiliated Hospital 2 of Nantong University and First People’s Hospital of Nantong City, No. 6 North Hai-Er-Xiang Road, Nantong, 226001 China; 3 Department of Nephrology, Affiliated Hospital 2 of Nantong University and First People’s Hospital of Nantong City, No. 6 North Hai-Er-Xiang Road, Nantong, 226001 China

**Keywords:** Nonalcoholic fatty liver disease, Platelet/high-density lipoprotein cholesterol ratio, Hepatic fibrosis, Inflammation, Hypercoagulability

## Abstract

The platelet/high-density lipoprotein cholesterol ratio (PHR) is a novel inflammatory and hypercoagulability marker that represents the severity of metabolic syndrome. Liver metabolic syndrome is manifested by nonalcoholic fatty liver disease (NAFLD), which is associated with inflammation and hypercoagulability. This cross-sectional investigation aimed to identify the relationship between PHR and NAFLD. Participants in the National Health and Nutrition Examination Survey (NHANES) 2017–2020 were evaluated for hepatic steatosis and fibrosis using vibration-controlled transient elastography. The PHR was calculated as the ratio of platelets to high-density lipoprotein cholesterol. Increased PHR was associated with an increased incidence of NAFLD and hepatic fibrosis. Compared with patients in the first PHR quartile, after adjustment for clinical variables, the corresponding odds ratio (OR) for NAFLD in the fourth quartile was 2.36 (95% CI, 1.76 to 3.18) (*p* < 0.05); however, the OR for hepatic fibrosis was not statistically significant (*p* > 0.05). Furthermore, restricted cubic spline analyses showed an S-shaped association between PHR and NAFLD and an L-shaped relationship between PHR and hepatic fibrosis. The results support the effectiveness of PHR as a marker for NAFLD and hepatic fibrosis. Therefore, interventions to improve the PHR may be of benefit in reducing the incidence of both hepatic steatosis and fibrosis.

## Introduction

Nonalcoholic fatty liver disease (NAFLD) is the most common chronic disorder of the liver, affecting approximately a quarter of the global population and posing a major threat to public health [1]. NAFLD is characterized by multiple pathological changes in the liver, of which benign simple hepatic steatosis is the least severe, nonalcoholic steatohepatitis (NASH) without fibrosis has minimal severity, and the most severe is NASH accompanied by fibrosis. Liver fibrosis results in irreversible structural changes that may eventually progress to hepatocellular carcinoma (HCC) [2, 3]. NAFLD is linked not only to poor liver prognosis but also to enhanced the risks of abnormal extrahepatic metabolism such as hyperlipidemia, hyperglycemia, hyperuricemia, and insulin resistance [4]. In combination with these metabolic abnormalities, NAFLD increases the patient's susceptibility to cardiovascular diseases and extrahepatic cancer, which account for the majority of deaths due to extrahepatic causes [5, 6]. Therefore, the identification of hepatic steatosis, fibrosis risk factors, and new therapeutic indices is urgently needed.

The synthesis of clotting factors is one of the liver’s major functions, and the literature suggests that NAFLD causes dysregulation of the clotting balance toward thrombosis [7, 8]. Hypercoagulability can aggravate NAFLD as fibrin can colocalize with proinflammatory macrophages in regions of hepatic steatosis [9]. Platelets are involved in hemostasis and promote coagulation. Fujimori et al. found that elevated or abnormal platelet counts are associated with hepatic fibrosis in NAFLD [10]. A case‒control investigation showed that the mean platelet volume representing platelet activation and activity is significantly associated with NAFLD [11]. NAFLD is also frequently accompanied by atherosclerotic dyslipidemia [12], suggesting elevated levels of triglyceride (TG) and low-density lipoprotein cholesterol (LDL-C) and decreased levels of high-density lipoprotein cholesterol (HDL-C) [13]. HDL-C facilitates the efflux of dietary cholesterol via the reverse cholesterol transport pathway and can exert anti-inflammatory and antioxidant effects [14]. Therefore, decreased HDL-C may contribute to NAFLD development due to reduced cholesterol efflux and antioxidant effects [15]. These results led to the speculation that the combination of platelet counts with HDL-C levels might have the potential to estimate the risks of NAFLD and hepatic fibrosis. Jialal et al. revealed that the platelet to HDL-C ratio (PHR) was significantly associated with the severity of metabolic syndrome [16]. As NAFLD is a manifestation of hepatic metabolic syndrome, it is possible that the PHR may be able to assess the severity of NAFLD. However, the relationships between the PHR and liver steatosis and fibrosis are unknown.

Thus, the present investigation used data on NAFLD from the National Health and Nutrition Examination Survey (NHANES) to investigate the association of PHR with hepatic steatosis and fibrosis in adults.

## Materials and methods

### Study participants

The NHANES (2017–2020) was carried out by the Centers for Disease Control and Prevention to acquire information about the general United States population’s nutrition and health using multistage, complex, and probabilistic sampling criteria [17]. A total of 15,560 participants were included in the 2017–2020 cycle, and 7,289 participants were selected for subsequent analyses after excluding underage individuals or individuals with missing vibration-controlled transient elastography (VCTE) results, hepatitis B antigen positivity, missing PHR data, hepatitis C antibody or RNA positivity, history of chronic hepatitis, and heavy alcohol consumption (> 4 drinks/day).

### Clinical variables

Demographic variables, including age, ethnicity, sex, family income to poverty threshold ratio, body mass index (BMI), smoking and drinking status, and past medical history of diabetes, hypertension, and coronary heart disease, were extracted from the NHANES database.

### Laboratory variables

Alanine aminotransferase (ALT), LDL-C, albumin (Alb), γ-glutamyltranspeptidase (GGT), HDL-C, aspartate aminotransferase (AST), TG, glycated hemoglobin (HbA1c), total cholesterol (TC), platelets (PLT) and fasting glucose were the laboratory variables included. The protocols for the measurement of these variables were obtained from the literature [18, 19]. The PHR was calculated as the ratio of PLT to HDL-C. Noninvasive tests (NITs) for liver steatosis include the Zhejiang University (ZJU) index [20] and hepatic steatosis index (HSI) [21], while noninvasive tests for liver fibrosis include the fibrosis-4 (FIB-4) and NAFLD fibrosis score (NFS) [22]. Their formulas are as follows: ZJU index = BMI + fasting glucose + TG + 3 × ALT/AST + 2 (if diabetic), HSI = 8 × ALT/AST + BMI + 2 (if diabetic) + 2 (if female), FIB-4 = age × AST/(PLT × √ALT) and NFS = -1.675 + 0.037 × age + 0.094 × BMI + 1.13 (if diabetic) + 0.99 × ALT/AST – 0.013 × PLT – 0.66 × Alb.

### Evaluation of hepatic steatosis and fibrosis

Experienced NHANES staff performed VCTE on each participant using the FibroScan®-equipped model 502 V2 Touch. The VCTE report reflected hepatic steatosis using the controlled attenuation parameter (CAP) and hepatic fibrosis by liver stiffness measurement (LSM). With reference to previous studies, NAFLD was diagnosed when CAP was ≥ 274 dB/m [23], with CAP ≥ 302 dB/m representing severe hepatic steatosis [23–25]. Hepatic fibrosis was graded into F2, F3, and F4, corresponding to thresholds of 8.2, 9.7, and 13.6 kPa, respectively [18, 26].

### Statistical analysis

The NHANES data were extracted, merged, cleaned, and analyzed by R software (version 4.2.2). *P* values < 0.05 were considered statistically significant. The participants were divided into four subgroups according to the PHR quartiles. For descriptive analyses, categorical variables are presented as frequencies (percentages), and normally and nonnormally distributed continuous variables are depicted as the mean ± SD and median (25 and 75% interquartile). To compare the differences in normally distributed, nonnormally distributed, and categorical data among the four subgroups, one-way analysis of variance (ANOVA), Kruskal‒Wallis, and chi-square tests, respectively, were conducted. Subsequently, Spearman correlation analyses were carried out to detect the correlations of the PHR with clinical variables. Multivariate linear regression analyses were conducted to identify the mean differences (B; 95% confidence interval [CI]) in CAP and lnLSM and the odds ratios (ORs [95% CI]) for NAFLD and hepatic fibrosis among the four subgroups, keeping the first quartile (Q1) as the standard reference. Finally, dose‒response relationships between PHR and NAFLD and hepatic fibrosis incidence were assessed using restricted cubic spline analyses (RCS). A *p* value for nonlinearity was assessed by testing the null hypothesis (stating: second spline coefficient = 0 [27]). The RCS models were set for sex, education level, age, household income, ethnicity, smoking status, diabetes and hypertension history, HbA1c, TG, TC, and LDL-C.

## Results

### Clinical characteristics of the study participants

As shown in Table 1, 7289 participants were included in the study. The average age of the participants was 48.75 ± 18.34 years, and the cohort included 49.2% males and 50.8% females. The prevalence of NAFLD and hepatic fibrosis was 42.6% and 9.5%, respectively. Increases in PHR were found to be associated with increased NAFLD prevalence, severe hepatic steatosis, hepatic fibrosis, CAP, LSM, severity of hepatic fibrosis, ZJU index and HSI (*p* < 0.05). Age, TC, sex, BMI, HDL-C, ethnicity, ALT, educational level, LDL-C, household income, GGT, smoking status, AST, diabetes, TGs, Alb, HbA1c, FIB-4 and NFS were significant variables in all PHR quartiles (all *p* < 0.05). There was no statistically significant difference in the PHR quartiles in terms of hypertension (*p* > 0.05).

**Table 1:** Clinical characteristics of the study participants

Variables	Total	Q1	Q2	Q3	Q4	*p* value
PHR	24.61–1923.08	< 139.69	139.69–182.18	182.18–234.10	> 234.10	
*n*	7289	1822	1822	1822	1823	
Age (years)	48.75 ± 18.34	53.86 ± 18.68	49.39 ± 18.71	47.72 ± 17.74	44.22 ± 16.89	< 0.001
Male, *n* (%)	3584(49.2)	812(44.6)	883(48.5)	955(52.4)	934(51.2)	< 0.001
BMI (kg/m^2^)	29.74 ± 7.41	26.84 ± 6.17	28.57 ± 6.60	30.79 ± 7.33	32.76 ± 8.02	< 0.001
Ethnicity, *n* (%)						< 0.001
Non-Hispanic White	2551(35.0)	676(37.1)	665(36.5)	616(33.8)	594(32.6)	
Non-Hispanic Black	1859(25.5)	516(28.3)	486(26.7)	432(23.7)	425(23.3)	
Mexican American	893(12.3)	159(8.7)	215(11.8)	253(13.9)	266(14.6)	
Other Race	1986(27.2)	471(0.259)	456(25.0)	521(0.286)	538(29.5)	
Education, *n* (%)						< 0.001
Less than high school	544(7.5)	115(6.3)	118(6.5)	162(8.9)	149(8.2)	
High school	783(10.7)	162(8.9)	173(9.5)	211(11.6)	237(13.0)	
More than high school	5962(81.8)	1545(84.8)	1530(84.0)	1449(79.5)	1437(0.788)	
Household income, *n* (%)						< 0.001
PIR < 1	1392(19.1)	304(16.7)	306(16.8)	368(20.2)	414(22.7)	
PIR 1 to < 3	3068(42.1)	743(40.8)	774(42.5)	776(42.6)	775(42.5)	
PIR ≥ 3	2829(38.8)	775(42.5)	742(40.7)	678(37.2)	634(34.8)	
Smoking status, *n* (%)	()	()	()	()	()	
Diabetes, *n* (%)	1011(13.9)	193(10.6)	228(12.5)	275(15.1)	315(17.3)	< 0.001
Hypertension, *n* (%)	2573(35.3)	647(35.5)	630(34.6)	645(35.4)	651(35.7)	0.903
TG (mmol/L)	1.24(0.87–1.83)	0.96(0.72–1.28)	1.14(0.84–1.61)	1.41(1.00–2.01)	1.64(1.15–2.48)	< 0.001
TC (mmol/L)	4.79 ± 1.04	4.93 ± 1.02	4.76 ± 1.02	4.76 ± 1.04	4.70 ± 1.07	< 0.001
HDL-C (mmol/L)	1.38 ± 0.41	1.79 ± 0.43	1.44 ± 0.27	1.25 ± 0.23	1.05 ± 0.22	< 0.001
LDL-C (mmol/L)	2.71(2.17–3.34)	2.56(2.10–3.23)	2.69(2.18–3.36)	2.85(2.28–3.44)	2.77(2.22–3.31)	< 0.001
ALT (U/L)	17(13–25)	16(12–22)	16(12–23)	18(13–27)	19(13–28)	< 0.001
AST (U/L)	19(16–23)	20(17–24)	19(16–23)	19(16–23)	18(15–23)	< 0.001
GGT (U/L)	20(14–31)	19(13–30)	19(13–28)	22(15–31)	23(16–34)	< 0.001
Alb (g/L)	40.76 ± 3.37	41.11 ± 3.28	40.92 ± 3.27	40.84 ± 3.31	40.18 ± 3.53	< 0.001
HbA1c (%)	5.81 ± 1.09	5.66 ± 0.89	5.74 ± 1.01	5.88 ± 1.14	5.96 ± 1.25	< 0.001
CAP (dB/m)	263.16 ± 62.80	241.84 ± 57.83	253.70 ± 60.26	272.19 ± 61.30	284.89 ± 62.87	< 0.001
LSM (kPa)	5.00(4.10–6.10)	4.80(4.00–5.90)	4.90(4.00–6.00)	5.00(4.10–6.20)	5.30(4.30–6.50)	< 0.001
NAFLD, *n* (%)	3108(42.6)	496(27.2)	662(36.3)	887(48.7)	1063(58.3)	< 0.001
Severe hepatic steatosis, *n* (%)	2000(27.4)	276(15.2)	261(14.3)	309(17.0)	318(17.4)	< 0.001
Hepatic fibrosis, *n* (%)	694(9.5)	132(7.2)	145(8.0)	205(11.3)	212(11.6)	< 0.001
F2, *n* (%)	259(3.6)	52(2.9)	57(3.1)	73(4.0)	77(4.2)	
F3, *n* (%)	238(3.3)	40(2.2)	42(2.3)	81(4.4)	75(4.1)	
F4, *n* (%)	197(2.7)	40(2.2)	46(2.5)	51(2.8)	60(3.3)	
ZJU index	40.88 ± 8.64	37.00 ± 7.04	39.27 ± 7.56	42.25 ± 8.56	45.01 ± 9.07	< 0.001
HSI	39.06 ± 8.81	35.17 ± 7.24	37.49 ± 7.84	40.53 ± 8.72	43.08 ± 9.20	< 0.001
FIB-4	0.87(0.55–1.32)	1.34(0.88–1.92)	0.96(0.65–1.35)	0.81(0.53–1.15)	0.58(0.40–0.84)	< 0.001
NFS	-1.84(-2.95–0.71)	-1.43(-2.56–0.42)	-1.80(-2.91–0.67)	-1.84(-2.92–0.71)	-2.34(-3.37–1.19)	< 0.001

Normally distributed values in the table are given as the mean ± SD, skewed distributed values are given as the median (25 and 75% interquartiles), and categorical variables are given as frequency (percentage)

*PHR* Platelet/high-density lipoprotein cholesterol ratio, *BMI* Body mass index, *PIR* Poverty income ratio, *TG* Triglyceride, *TC* Total cholesterol, *HDL-C* High-density lipoprotein cholesterol, *LDL-C* Low-density lipoprotein cholesterol, *ALT* Alanine aminotransferase, *AST* Aspartate aminotransferase, *GGT* γ-glutamyltranspeptidase, *Alb* Albumin, *HbA1c* Glycosylated hemoglobin A1c, *CAP* Controlled attenuation parameter, *LSM* Liver stiffness measurement, *NAFLD* Nonalcoholic fatty liver disease, *ZJU index* Zhejiang University index, *HSI* Hepatic steatosis index, *FIB-4* Fibrosis-4, *NFS* Nonalcoholic fatty liver disease fibrosis score

### Relationships between PHR and clinical variables

As illustrated in Table 2, PHR was positively associated with BMI, TG, LDL-C, ALT, GGT, HbA1c, CAP, LSM, ZJU index and HSI (*r* = 0.335, 0.400, 0.069, 0.119, 0.128, 0.120, 0.277, 0.107, 0.383 and 0.368, respectively, *p* < 0.05) and negatively associated with age, TC, AST, Alb, FIB-4 and NFS (*r* = -0.190, -0.084, -0.088, -0.095, -0.495 and -0.184, respectively, *p* < 0.05).

**Table 2:** Relationships between PHR and clinical parameters

Variables	*r*	*P* value
Age	-0.190	< 0.001
BMI	0.335	< 0.001
TG	0.400	< 0.001
TC	-0.084	< 0.001
LDL-C	0.069	< 0.001
ALT	0.119	< 0.001
AST	-0.088	< 0.001
GGT	0.128	< 0.001
Alb	-0.095	< 0.001
HbA1c	0.120	< 0.001
CAP	0.277	< 0.001
LSM	0.107	< 0.001
ZJU index	0.383	< 0.001
HSI	0.368	< 0.001
FIB-4	-0.495	< 0.001
NFS	-0.184	< 0.001

*r* Spearman’s correlation coefficient

*PHR* Platelet/high-density lipoprotein cholesterol ratio, *BMI* Body mass index, *TG* Triglyceride, *TC* Total cholesterol, *LDL-C* Low-density lipoprotein cholesterol, *ALT* Alanine aminotransferase, *AST* Aspartate aminotransferase, *GGT* γ-glutamyltranspeptidase, *Alb* Albumin, *HbA1c* Glycosylated hemoglobin A1c, *CAP* Controlled attenuation parameter, *LSM* Liver stiffness measurement, *NAFLD* Nonalcoholic fatty liver disease, *ZJU* Index Zhejiang University index, *HSI* Hepatic steatosis index, *FIB-4* Fibrosis-4, *NFS* Nonalcoholic fatty liver disease fibrosis score

### Multivariate regression analysis of CAP and LSM in the PHR quartiles

After adjustment of the clinical variables, the adjusted mean difference (B) in the CAP of the participants in PHR Q4 versus Q1 was 22.60 dB/m (95% CI, 15.51 to 29.70). However, (B) in LSM was not statistically significant (*p* > 0.05) (Table 3).

**Table 3:** Mean differences (B [95% CI]) in CAP and LSM among the quartiles of PHR

PHR quartiles	Model 0	*P* value	Model 1	*P* value	Model 2	*P* value	Model 3	*P* value
CAP, dB/m								
Q1	0-reference	-	0-reference	-	0-reference	-	0-reference	-
Q2	11.86(7.93 to 15.80)	< 0.001	14.83(10.98 to 18.68)	< 0.001	12.42(8.58 to 16.25)	< 0.001	6.63(1.03 to 12.22)	0.02
Q3	30.35(26.41 to 34.28)	< 0.001	34.22(30.33 to 38.11)	< 0.001	30.05(26.17 to 33.92)	< 0.001	17.71(11.53 to 23.89)	< 0.001
Q4	43.05(39.12 to 46.99)	< 0.001	48.67(44.72 to 52.62)	< 0.001	43.20(39.23 to 47.18)	< 0.001	22.60(15.51 to 29.70)	< 0.001
LnLSM, kPa								
Q1	0-reference	-	0-reference	-	0-reference	-	0-reference	-
Q2	0.014(-0.013 to 0.040)	0.308	0.023(-0.004 to 0.049)	0.095	0.011(-0.015 to 0.038)	0.406	-0.044(-0.083 to -0.004)	0.032
Q3	0.063(0.037 to 0.040)	< 0.001	0.077(0.050 to 0.104)	< 0.001	0.053(0.026 to 0.080)	< 0.001	-0.006(-0.050 to 0.038)	0.800
Q4	0.104(0.077 to 0.130)	< 0.001	0.122(0.095 to 0.149)	< 0.001	0.091(0.064 to 0.119)	< 0.001	-0.007(-0.058 to 0.043)	0.779

Model 0: unadjusted model

Model 1: adjusted for age, sex, ethnicity, education, PIR, smoking status

Model 2: additionally adjusted for diabetes and hypertension

Model 3: additionally adjusted for HbA1c, TG, TC, LDL-C

### Multivariate analysis of factors influencing NAFLD and hepatic fibrosis according to the PHR quartiles

In comparison with participants in Q1, those in Q2, Q3, and Q4 showed NAFLD ORs of 1.52 (95% CI, 1.32 to 1.76), 2.53 (95% CI, 2.21 to 2.91), and 3.74 (95% CI, 3.25 to 4.30), respectively (*p* < 0.05) (Table 4). Furthermore, after adjusting for other clinical variables via multivariate logistic regression analysis, the corresponding NAFLD ORs of Q2, Q3, and Q4 versus the PHR values of participants in Q1 were 1.44 (95% CI, 1.14 to 1.83), 1.95 (95% CI, 1.50 to 2.52), and 2.36 (95% CI, 1.76 to 3.18), respectively (all *p* < 0.05). In contrast with the participants in Q1 of the PHR, the ORs of hepatic fibrosis for participants in Q2, Q3, and Q4 were 1.11 (95% CI, 0.87 to 1.42) (*p* > 0.05), 1.62 (95% CI, 1.29 to 2.04) (*p* < 0.05), and 1.68 (95% CI, 1.34 to 2.12) (*p* < 0.05), respectively. However, after adjusting for other clinical variables, the ORs of hepatic fibrosis became nonsignificant (*p* > 0.05) (Table 4).

**Table 4:** ORs (95% CIs) of NAFLD and hepatic fibrosis according to the quartiles of PHR

PHR quartiles	Model 0	*P* value	Model 1	*P* value	Model 2	*P* value	Model 3	*P* value
NAFLD								
Q1	1-reference	-	1-reference	-	1-reference	-	1-reference	-
Q2	1.52(1.32–1.76)	< 0.001	1.68(1.45–1.95)	< 0.001	1.68(1.45–1.95)	< 0.001	1.44(1.14–1.83)	0.003
Q3	2.53(2.21–2.91)	< 0.001	2.99(2.58–3.47)	< 0.001	2.99(2.58–3.47)	< 0.001	1.95(1.50–2.52)	< 0.001
Q4	3.74(3.25–4.30)	< 0.001	4.75(4.08–5.54)	< 0.001	4.75(4.08–5.54)	< 0.001	2.36(1.76–3.18)	< 0.001
Hepatic fibrosis								
Q1	1-reference	-	1-reference	-	1-reference	-	1-reference	-
Q2	1.11(0.87–1.42)	0.417	1.22(0.95–1.57)	0.116	1.22(0.95–1.57)	0.116	0.77(0.52–1.13)	0.180
Q3	1.62(1.29–2.04)	< 0.001	1.84(1.45–2.34)	< 0.001	1.84(1.45–2.34)	< 0.001	0.89(0.59–1.34)	0.570
Q4	1.68(1.34–2.12)	< 0.001	2.00(1.57–2.55)	< 0.001	2.00(1.57–2.55)	< 0.001	0.66(0.41–1.06)	0.088

Model 0: unadjusted model

Model 1: adjusted for age, sex, ethnicity, education, PIR, smoking status

Model 2: additionally adjusted for diabetes and hypertension

Model 3: additionally adjusted for HbA1c, TG, TC, LDL-C

### Dose‒response relationships between PHR and NAFLD and hepatic fibrosis

**Fig. 1:**
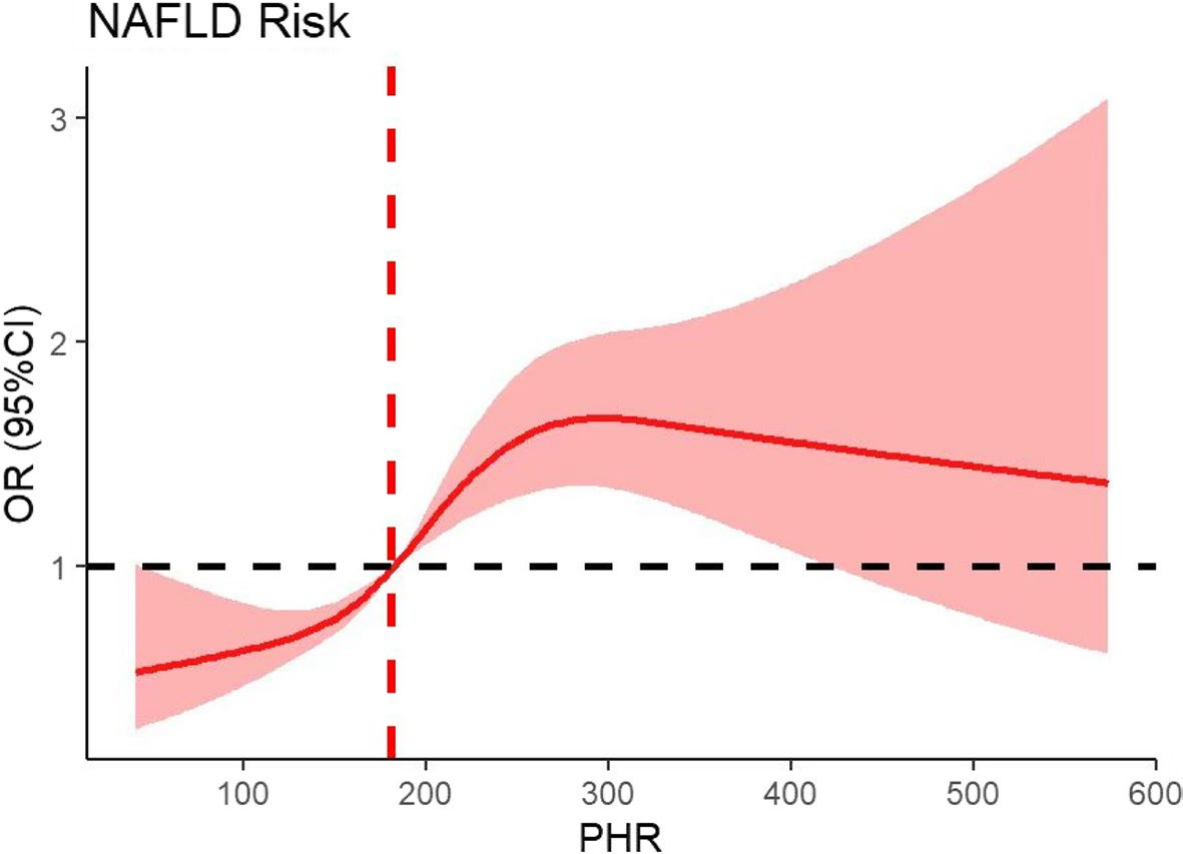
The nonlinear relationship between the PHR and the risk of NAFLD. A nonlinear relationship was detected after adjusting for sex, education level, age, household income, ethnicity, smoking status, diabetes and hypertension history, HbA1c, TG, TC, and LDL-C

**Fig. 2:**
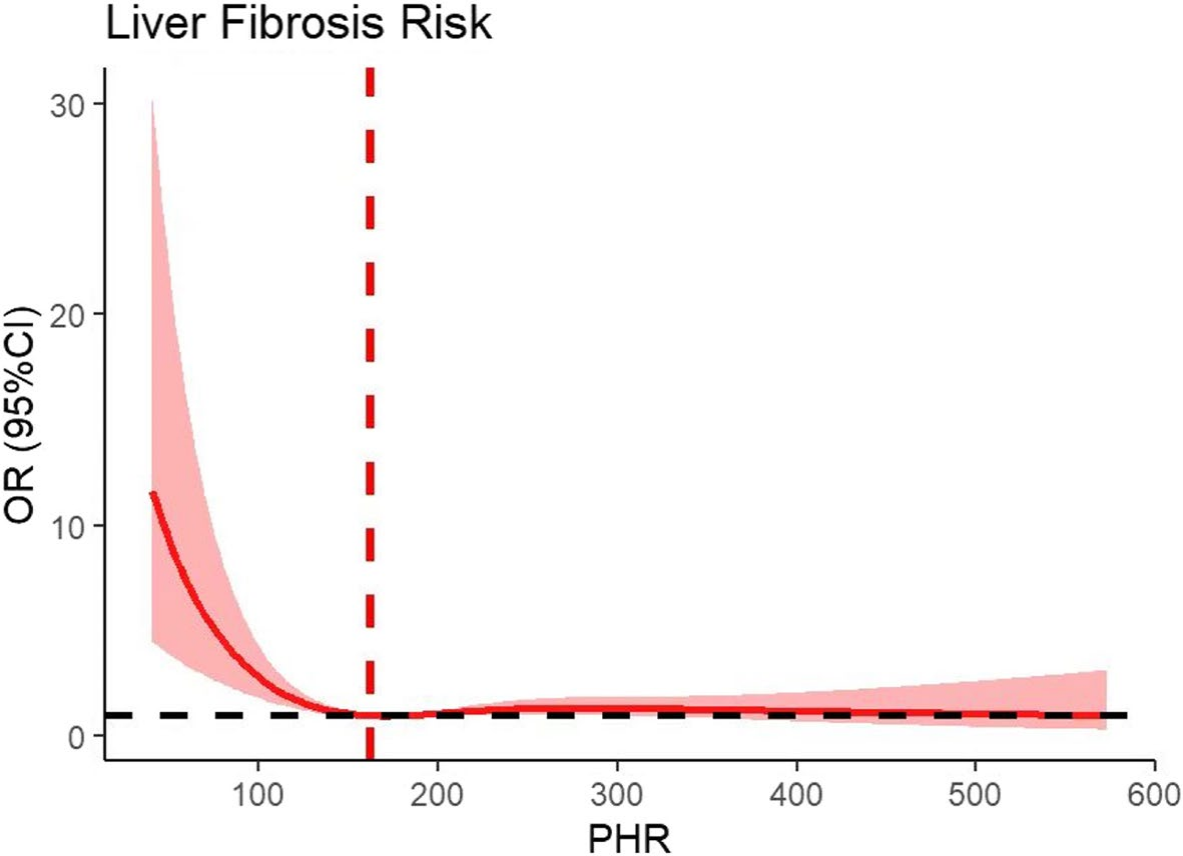
The nonlinear relationship between the PHR and the risk of liver fibrosis. A nonlinear relationship was detected after adjusting for sex, education level, age, household income, ethnicity, smoking status, diabetes and hypertension history, HbA1c, TG, TC, and LDL-C

As shown in Figs. 1 and 2, the PHR was nonlinearly linked with the prevalence of NAFLD and hepatic fibrosis (*p* for nonlinearity < 0.01). Figure 1 reveals that at PHR < 181, the smaller the PHR, the lower the risk of NAFLD. In Fig. 2, the RCS curve indicated that at PHR = 162 (reference), a lower PHR level was related to a higher risk of hepatic fibrosis.

## Discussion

This investigation evaluated the associations between PHR and NAFLD and hepatic fibrosis in US adults from the NHANES. It is worth noting that there was an S-shaped association between PHR and NAFLD and an L-shaped association between PHR and hepatic fibrosis, indicating that an appropriate PHR range (162–181) might be beneficial for the assessment of both hepatic steatosis and fibrosis.

Liver biopsy is the gold standard procedure for NAFLD diagnosis and severity of hepatic fibrosis; however, its application is limited due to various reasons such as high cost, invasiveness, and risks of complications [28]. Noninvasive detection methods include elastography techniques based on ultrasound and magnetic resonance imaging, but the high cost and relative inaccessibility of magnetic resonance spectroscopy (MRS) limit its clinical application; therefore, VCTE is the most widely used evaluation method globally [29]. The literature indicates that the results of CAP and LSM obtained from VCTE grade hepatic steatosis and fibrosis are accurately, comparable to liver biopsy [30, 31]. Therefore, the conclusions obtained by investigating the relationships between the PHR and VCTE results were accurate.

NAFLD is characterized by fatty acid deposition, lipotoxic lipid responses, inflammation, fibrogenesis, oxidative stress, insulin resistance, and microbial disorders [32]. Metabolic syndrome is the strongest risk factor for NAFLD, and their association may be bidirectional [32]. Previous research has confirmed a link between PHR and metabolic syndrome, which might be mediated by inflammation and procoagulant diathesis, and they also observed that the PHR was positively associated with systolic and diastolic blood pressure, fasting glucose and homeostasis model assessment of insulin resistance (HOMA-IR) [16]. Hypertension and type 2 diabetes (T2D) are both important risk factors for NAFLD [33], and observational studies revealed that the proportion of NAFLD combined with hypertension and T2D was as high as 70% [34]. Activated platelets can secrete multiple mediators involved in the onset of hypertension [35], and low levels of HDL-C might indirectly increase the secretion of aldosterone and lead to hypertension [36]. Hence, it was reasonable that the PHR was closely related to hypertension. Platelet count is an indicator of inflammation, while HDL-C exerts an anti-inflammatory effect, so PHR may be a surrogate of inflammation and is closely related to the status of T2D. Therefore, hypertension and T2D may be intermediate mediators between PHR and NAFLD.

After four days of feeding mice a high-fat diet (HFD), histological evaluation of the livers showed significantly increased expression of pro-inflammatory cytokines and pro-coagulation factors, indicating that HFD promotes hepatic steatosis by inducing inflammation and hypercoagulability [37]. Multiple epidemiological studies have demonstrated a close association between inflammation and NAFLD progression [38–40]. The sera of patients with histologically verified NAFLD also showed features associated with mild systemic inflammation [41]. Another study based on the NHANES 2017–2020 indicated a strong correlation between the systemic immune-inflammation index and hepatic steatosis; however, no significant correlation was reported with hepatic fibrosis [23]. A multicenter cohort study showed that lobular inflammation on liver biopsy was a feature of possible NAFLD progression [42]. Therefore, inflammation partially explains the link between PHR and NAFLD.

Platelets promote coagulation, and HDL-C both inhibits platelet reactivity and stimulates clot fibrinolysis [43]. Therefore, it can be speculated that the PHR is closely related to the coagulation status of an individual. The relationship of NAFLD with hypercoagulability is also bidirectional. On the one hand, the liver is the primary site of clotting-factor synthesis and as revealed by a single-center cohort study, NAFLD severity is independently responsible for the elevation of plasminogen activator inhibitor-1 (PAI-1) levels [44]. Upregulation of PAI-1 can inhibit the fibrinolysis system, thereby promoting fibrin accumulation and thrombus formation [45]. On the other hand, hypercoagulability may promote liver disease by inducing fibrosis, blocking liver vessels, and causing loss of the liver parenchyma [46]. An in vitro investigation showed that heparin, a commonly used anticoagulant, could prevent liver necrosis by inhibiting hypercoagulability induced by HFD [47]. In addition to participating in the inflammatory response and coagulation, platelets also play a role in activating the immune system. Platelets can interact with hyaluronic acid in the extracellular matrix of hepatocytes through the CD44 receptor to accumulate in the damaged liver, activate T cells in the liver parenchyma, and ultimately aggravate hepatic steatosis [48]. Therefore, a high PHR level may be an inflammatory and hypercoagulation marker and may be closely related to hepatic steatosis severity.

Interestingly, compared with the marked positive correlation of PHR with NAFLD, the present study showed that lower PHR levels were associated with an increased risk of hepatic fibrosis after adjustment for clinical variables. FIB-4 and NFS increased with worsening hepatic fibrosis, while we observed a decreasing trend of FIB-4 and NFS with increasing PHR quartiles and significant negative correlations between PHR and FIB-4 and NFS, which also confirmed that the increase in PHR to a certain extent was closely related to the decrease in the risk of hepatic fibrosis. A large retrospective cohort study in Japan observed that the platelet counts of NAFLD patients decreased linearly with increasing severity of liver fibrosis [49]. Similarly, in another study that included patients with NAFLD and chronic liver disease caused by hepatitis C virus infection, the platelet counts declined progressively with the severity of hepatic fibrosis [50]. Retrospective analyses of hepatic cirrhosis patients revealed that platelet counts declined progressively over 15 years prior to disease onset [51]. Platelet production is primarily regulated by thrombopoietin synthesized in the liver. Along with the progression of NAFLD, excessive lipid deposition and oxidative stress may damage mitochondrial function in liver cells, adversely affecting the synthesis of thrombopoietin and eventually leading to reduced platelet counts [52, 53]. These may be one of the mechanisms underlying the association of PHR levels with hepatic fibrosis. In addition, decreased HDL-C is recognized as a hallmark of hepatic fibrosis and is associated with poor prognosis in patients with chronic liver disease [54]. The follow-up of NAFLD patients with liver fibrosis showed that low HDL-C increased the risk of progression to hepatocellular carcinoma [14]. Therefore, we speculate that this is why the risk of liver fibrosis does not decrease further as the PHR increases.

## Study strengths and limitations

The strengths of this study are the large sample size and the relatively reliable assessments of hepatic steatosis and fibrosis. However, the study has several limitations. First, due to its observational cross-sectional design, it was unable to elucidate a causal relationship between PHR and hepatic steatosis and fibrosis. Longitudinal and interventional studies are required to address this limitation. Second, due to the limitations of the NHANES database, some confounding factors that may have had some influence on the results were not adjusted. Third, liver biopsy is the gold standard test for grading hepatic steatosis and fibrosis, and although transient elastography, as used here, is extremely accurate, it is still subtly different from liver biopsy. Fourth, we also evaluated the associations between the PHR and several NITs but could not conclude that the PHR was superior to these NITs. However, the PHR is simpler to calculate than these indices and is closely related to both hepatic steatosis and fibrosis. In the future, studies are needed to verify the conclusions of this investigation and address the aforementioned limitations.

## Conclusions

In conclusion, the results showed a significant nonlinear association between PHR and hepatic steatosis and fibrosis and suggested that PHR can be used as a potential marker for hepatic steatosis and fibrosis and, in an appropriate range, might be of benefit to improve these conditions.

## Data Availability

The National Health and Nutrition Examination Survey dataset is publicly available at the National Center for Health Statistics of the Centers for Disease Control and Prevention (https://wwwn.cdc.gov/nchs/nhanes/nhanes3/datafiles.aspx).
